# Vitamin D, Calcium, Parathyroid Hormone, and Sex Steroids in Bone Health and Effects of Aging

**DOI:** 10.1155/2020/9324505

**Published:** 2020-06-17

**Authors:** Hitesh Kumar Bhattarai, Shreya Shrestha, Kabita Rokka, Rosy Shakya

**Affiliations:** Department of Biotechnology, Kathmandu University, Dhulikhel, Nepal

## Abstract

Bone health of the elderly is a major global health concern, since about 1 in 3 women and 1 in 5 men suffer from bone loss and fractures, often called osteoporosis, in old age. Bone health is a complex issue affected by multiple hormones and minerals. Among all the hormones involved in bone health, calcitriol (also vitamin D), parathyroid, and sex hormones (especially estrogen) have been discussed in this review paper. We have discussed the metabolism of these hormones and their effects on bone health. Vitamin D can be obtained from diet or formed from 7-dehydrocholesterol found under the skin in the presence of sunlight. The active form, calcitriol, causes dimerization of vitamin D receptor and acts on the bones, intestine, and kidney to regulate the level of calcium in blood. Similarly, parathyroid hormone is secreted when the serum level of calcium is low. It helps regulate the level of blood calcium through calcitriol. Sex hormones regulate bone modeling at an early age and remodeling later in life. Loss of ovarian function and a decrement in the level of production of estrogen are marked by bone loss in elderly women. In the elderly, various changes in the calcium and vitamin D metabolism, such as decrease in the production of vitamin D, decrease in dietary vitamin D, decreased renal production, increased production of excretory products, decrease in the level of VDR, and decreased calcium absorption by the intestines, can lead to bone loss. When the elderly are diagnosed with osteoporosis, medications that directly target bone such as bisphosphonates, RANK ligand inhibitors, estrogen and estrogen analogues, estrogen receptor modulators, and parathyroid hormone receptor agonists are used. Additionally, calcium and vitamin D supplements are prescribed.

## 1. Bone Health

Bone is a very important organ that not only provides structural support and mobility but also acts as a storehouse of the minerals such as calcium and phosphorous. Strength of the bone is a factor of genetics as well as use. To keep bones strong, constant mechanical pressure has to be applied to bones. Bones are made up of minerals and proteins, primarily, both of which are important in conferring properties to bone. Minerals alone would make the bone too brittle and proteins alone would make it too soft and flexible. The minerals, calcium and phosphorous, are deposited as crystals of hydroxyapatite in the matrix made up of collagen. Collagen is produced by bone cells [1].

Bone renewal is generally described as two processes: remodeling and modeling [1]. When the site of bone formation and bone degradation varies, the renewal of bones is called modeling. The bone modeling process occurs early in the life, during childhood and adolescence, during which bone grows in size and shifts in space. Osteoclasts and osteoblasts are two major bone cells involved in the bone renewal process including modeling. Osteoclasts lead to reabsorption of the bones, while osteoblasts lead to formation of new bone tissues. Osteoclasts and osteoblasts act in different locations during the modeling process. During the remodeling process, osteoclasts and osteoblasts act in the same space leading to renewal of bones without shifting in space. The remodeling process is active throughout the life in humans and is important for various reasons. It leads to repair of fractures and cracks that occur in the bones, renewal of old bone cells that could cause bone cells to become brittle, and release of calcium from bone tissues [1]. During the remodeling process, bone formation is almost inevitably followed by bone resorption (i.e., the process of bone resorption by osteoclasts takes places before osteoblasts come into play) [2].

With aging, the pathophysiology of bone loss in male and females is slightly different. In elderly women, there is an increase in the rate of bone remodeling process and also a negative remodeling balance (meaning resorption takes place more than formation). This leads to bone loss and disruption of the microarchitecture of the bone. In elderly men, aging causes a reduction in bone formation and leads to low bone turnover [3–5].

**Figure 1:**
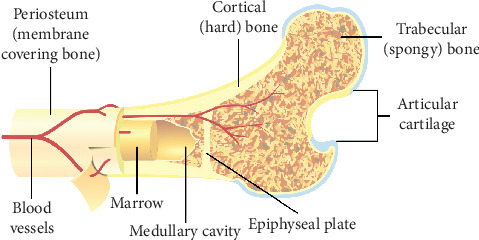
General structure of a bone (https://commons.wikimedia.org/wiki/User:Pbroks13). Two of the most important components of the bone are the trabecular component and the cortical component. The trabecular component is the spongy part of the bone that first absorbs shock. Shock is then transferred onto cortical component.

Major bones of the body can be divided into two components: the trabecular component and the cortical component (Figure 1). Making up about 80% of the bone, cortical component has low surface area to volume ratio. Vasculature occupies about 30% of this component. Aging causes porosity of the cortical component, leading to increased surface area. Twenty percent of the trabecular component is composed of bone tissues, and the rest are composed of marrow and fat. The function of the trabecular component is to transfer mechanical load to the cortical component. The trabecular component is usually used to absorb shock (Figure 1). Generally, the trabecular component has less mineral deposits and more water content than the cortical component. Additionally, the trabecular component is more exposed to the vasculature system and the turnover for this component is higher. Along the surface of the trabecular bone, resorption takes place. For resorption of the cortical component, tunneling through the bone has to take place [6].

A number of hormones, calcitriol, parathyroid hormone (PTH), calcitonin, sex hormones (estrogen and testosterone), growth hormones, thyroid hormones, cortisol, and insulin, affect bone health. Among these hormones, calcitriol, parathyroid hormone, and sex hormones will be discussed in detail since they are important in bone health in the elderly.

## 2. Vitamin D Metabolism

**Figure 2:**
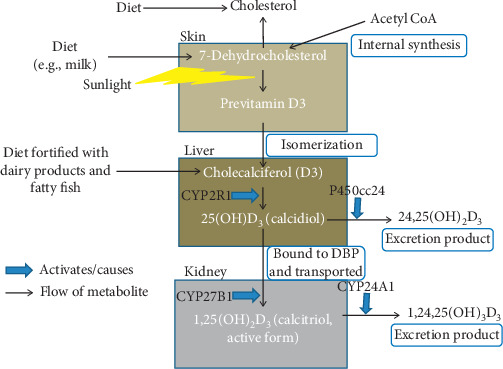
Vitamin D metabolism. The production of vitamin D starts from 7-dehydrocholesterol, which is found in abundance in the skin. It can be formed from internal synthesis pathway of cholesterol formation from acetyl CoA. In the pathway, it is formed before cholesterol. It can also be obtained from diet such as milk. 7-Dehydrocholesterol is converted to previtamin D3 by breaking one of the rings using sunlight. Previtamin D3 isomerizes to form cholecalciferol (or vitamin D3), which is hydroxylated using CYP2R1 enzyme in the liver to form 25(OH)D_3_ (or calcidiol). Vitamin D3 can also be obtained from diet fortified with dairy products or fatty fish. Calcidiol can be converted to 24,25(OH)_2_D_3_ by P450cc24 and excreted. From the liver, calcidiol is bound to vitamin D binding protein (DBP) and transported to kidneys where it is converted to 1,25(OH)_2_D_3_ (or calcitriol) by the enzyme CYP27B1. Calcitriol is the active form of vitamin D. Its role will be described in Figure 3. Excess calcitriol is converted to 1,24,25(OH)_3_D_3_ by CYP24A1 and excreted.

The term “vitamin D” originally described a trace substance that cured rickets in dogs. Dogs restricted indoors developed rickets and could be cured by feeding cod liver oil [7]. So, vitamin D was described as the substance found in cod liver oil [8]. Nowadays, vitamin D commonly denotes the chemicals cholecalciferol (abbreviated as D_3_) and ergocalciferol (abbreviated as D_2_). D_3_ is a secosteroid formed when the steroid nucleus of 7-dehydrocholesterol, formed from cholesterol, is broken down at the B ring by the action of UV rays from sunlight (Figure 2). D_3_ is either directly formed by the action of sunlight or can form indirectly from previtamin D_3_, also called tachysterol, which is predominantly formed when 7-dehydrocholesterol is exposed to sunlight (Figure 2). Since plenty of 7-dehydrocholesterol is found under the skin of humans and higher animals, sunlight is often the limiting factor for D_3_ formation.

In individuals, vitamin D can be obtained either from the sunlight or from dietary intake of food rich in vitamin D such as fatty fish (Figure 2). For the activation of vitamin D, two hydroxylation steps are required. The first step occurs in the liver where 25 hydroxy D_3_ (25(OH)D_3_) is produced by 25-hydroxylase (CYP2R1). The next step occurs mainly in the kidney, which involves the formation of 1,25 dihydroxy D_3_ (1,25(OH)_2_D_3_) from hydroxylation by 1 *α*-hydroxylase (CYP27B1) (Figure 2). Both 1,25(OH)_2_D_3_) and 25(OH)D_3_ are carried in the circulation by vitamin D binding protein (DBP). The active component of vitamin D is 1,25(OH)_2_D_3_, which binds to vitamin D receptor (VDR) and activates calcium metabolism, and is described later. The enzyme P450cc24 (CYP24 hydroxylase) converts 25(OH)D_3_ to the nonactive metabolite, 24,25(OH)_2_D_3_, in the kidney, thereby limiting the production of 1,25(OH)_2_D_3_. Similarly, 1,25(OH)_2_D_3_ is converted to 1,24,25(OH)_2_D_3_, an excretion product, by CYP24A1. Therefore, both the production and excretion of the active constituent of vitamin D can be controlled to keep its level constant (Figure 2) [9].

**Figure 3:**
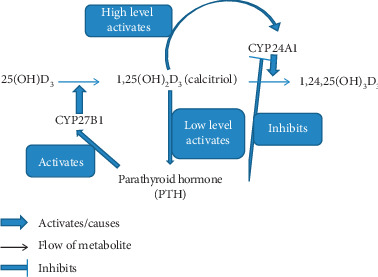
Autoregulation of calcitriol level. Calcitriol, the active form of vitamin D, is capable of autoregulating its level. When high level of calcitriol is found in the blood, it activates the enzyme CYP24A1, which converts it into excretion product. Low level of calcitriol stimulates production of parathyroid hormone. Parathyroid hormone has two-fold activity: it stimulates CYP27B1 enzyme to produce more of calcitriol from calcidiol and it inhibits the activity of CYP24A1, which does not let calcitriol convert to excretion product.

Calcitriol also regulates its own level through two mechanisms. Higher level of calcitriol activates CYP24A1 production leading to conversion of calcitriol to excretion product (Figure 3). On the contrary, low level of calcitriol stimulates secretion of parathyroid hormone, which on the one hand stimulates the production of calcitriol and on the other hand inhibits the production of excretion product (Figure 3).

Before delving into detail about the different pathways dysregulated in vitamin D metabolism in the elderly, here are some of the immediate factors that could lead to vitamin D deficiency in the diet in the elderly: (a) lack of sunlight leading to lower vitamin D3 level, (b) avoidance of dairy vitamin D due to lactase deficiency, and (c) improper absorption of vitamin D from the intestines [10].

## 3. Parathyroid Hormone and Calcium Metabolism

Parathyroid glands secrete parathyroid hormone and are a collection of four glands found adjacent to the thyroid gland. Parathyroid hormone (PTH) is a peptide hormone initially secreted as a 115 amino acid long pre-pro-PTH. Pre-pro-PTH is cleaved twice to form 84 amino acid long PTH. The hormone is secreted when the levels of calcium or calcitriol in serum is low. We have discussed in the previous section how PTH restores level of calcitriol. PTH acts on three organs, kidneys, intestine, and bone, to increase serum calcium level. In the kidneys, calcium and PTH receptors overlap; hence, PTH has direct effect on calcium metabolism. Increased calcium is extracted from glomerular filtrate in the kidney upon PTH action. In the bones, PTH has both catabolic and anabolic effects. The effect on bone can be classified into early effect, which leads to release of calcium in the serum from bones, and late effect, which includes reabsorption and bone remodeling. Among the two types of bone cells, osteoblasts seem to interact with PTH hormone. Osteoclasts may lack PTH receptors [11]. PTH promotes calcium absorption from the bone indirectly through the action of calcitriol.

## 4. Sex Hormone and Bone Metabolism

Sex hormones, estrogens and androgens, play a key role in bone metabolism. Earlier, it was hypothesized that estrogens inhibit bone formation, whereas androgens stimulate bone formation leading to higher bone density in males than in females. Additionally, it was hypothesized that men do not lose as much bone as women at old age because women suffer from menopause, which leads to a loss of estrogen. However, more recently, it has been discovered that androgens can be converted to estrogens, and bone contains receptors for both estrogens and androgens. So, there is no such thing as male-specific and female-specific bone metabolism by these hormones [12].

Sex steroids (androgens and estrogens) are produced by the adrenal cortex and male and female gonads, testis and ovary. Androgens are produced by the adrenal cortex in both males and females and by testes in men. The most important androgens are testosterone (T) and 5*α*-dihydrotestosterone (DHT) [13]. Most of T in males is secreted by testes. T acts directly to androgen receptor (AR) or indirectly to AR through conversion to DHT by 5*α*-reductase [13]. T is converted by P450 aromatase to estradiol (E2), which binds to estrogen receptors *α* and *β* (ER*α* and ER*β*). Adrenal androgens can be converted to estrone (E1) by aromatase or to T [14]. Only about 20 percent of E2 is produced by the testes in males; the rest is derived from androgens in peripheral tissues. The activity of ER can be controlled by controlling the amount of T, androgen production, and E2 conversion locally. In men and women, the pathway of estrogen and androgen production is similar. In females, since there is no testis, the ovaries produce androgens and convert them to estrogens. About 95 percent of E2 is secreted by the ovaries in women before menopause. After menopause, all the estrogens in females are derived from adrenal androgens, such that estrogen levels in postmenopausal women are less than those of men. In aging men, T levels decrease marginally, whereas E2 level stays more or less constant. The bioavailability of androgen and estrogen depends on another factor: sex hormone binding globulin (SHBG). In men, about 50–60 percent of T is bound to SHBG and is not bioavailable. This amount increases in the elderly men. This factor is not as important in elderly women as less SHBG binds to E2 [12].

ER*α* and ER*β* and AR are found in the bone cells, osteoblasts, osteoclasts, and osteocytes [15]. Hormone binding and signaling can take the form of genomic DNA activation through receptors in the cytosol or a more direct activation through membrane receptor [12].

In both males and females, bone development (modeling) is closely related to the production of sex steroids and expression of sex steroid receptors in the bones. While both male and female bones are enlarged during puberty, males experience higher degree of bone growth than females [16]. Besides development during puberty, sex steroids and receptors are important throughout life in remodeling bones. Loss of androgens in males retards bone development as observed in hypogonadal men [17]. Similarly, in postmenopausal women, estrogen level falls off and can lead to loss of bone mineral density and osteoporosis.

An important question is how estrogen influences bone remodeling. The result of gonadal insufficiency has been studied in bone marrow cocultures. Osteoclasts are an important class of bone cells described earlier that lead to bone resorption. Estrogens decrease the number and activity of osteoclasts and thus lead to loss of resorption. Estrogens act on different cell types such as monocytes, marrow stromal cells, and osteoblasts and lead to regulation of cytokines produced by these cells such that the number and activity of the osteoclasts decrease [18]. Estrogen causes monocytes to decrease the production of cytokines IL-1 and TNF-*α*, marrow stromal cells and osteoblasts to decrease the production of IL-6, GM-CSF, M-CSF, and RANKL, and increased production of OPG and osteoblasts to increase the production of TGF*β*, which has the desired effect of decreasing the number and activity of osteoclasts. Similarly, estrogens can directly lead to reduction in apoptosis of osteoclasts [12].

## 5. Calcium Metabolism

Calcium and phosphorous are two metals that are found as deposits in the bone. Among the two, calcium metabolism is very intricately linked to hormone metabolism and aging and will be discussed in the paper. Among the different minerals found in the body, calcium is the fifth most abundant one [19]. Human body contains about 1.2 kg (equivalent to about 300 mmol) of calcium, most of which is found in bones and teeth [19]. The calcium mineral has two major roles to play in the body: maintenance of structural integrity and regulation of metabolic function [20].

Absorption of calcium taken in through the diet primarily takes place in the jejunum but also in the ilium and colon. The absorption can be active (requiring energy) or passive. When calcium concentration is low, active transport is necessary, but as calcium concentration increases, passive diffusion comes into play. Usually, active transport of calcium across the intestinal cells is stimulated by calcitriol by producing calcium binding protein [21]. The activation of active transport by calcitriol in the villi cells takes place through normal hormonal signaling pathway: binding of the receptor, DNA interaction, and subsequent messenger RNA production. Calcitriol activity is critical in the maintenance of calcium absorption and skeletal integrity. Negative calcium balance, secondary hyperparathyroidism (when calcium absorption is low), increased bone loss, and osteoporosis all result from malabsorption of calcium. Although it will be discussed in more detail later, it would be imperative to mention that aging causes decline in calcium absorption. This will be observed after the age of 60 years, and after 80 years, almost everyone shows decline in calcium absorption [22].

**Figure 4:**
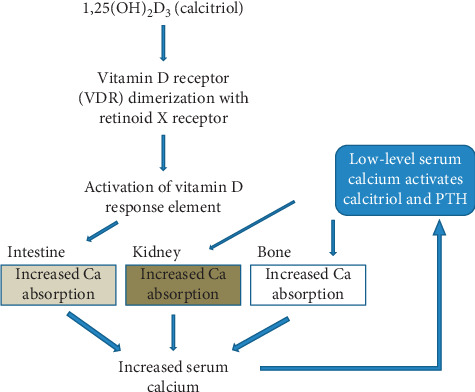
Effect of calcitriol on serum calcium level. Calcitriol, the active form of vitamin D, causes vitamin D receptor (VDR) to dimerize with retinoid X receptor. This causes activation of vitamin D response element in the genome. In the intestine, this further results in increased absorption of calcium from food and leads to increased serum calcium level. When the serum calcium level drops, calcitriol and PTH are activated. This causes increased calcium absorption from the kidneys and bone, where there is largest deposit of calcium. This absorption again leads to increased serum calcium.

Calcitriol (1,25(OH)₂D_3_) acts through the VDR and synthesizes genes and proteins involved in calcium transport (Figure 4) [23]. Deletion of the VDR results in malabsorption of calcium. VDR contains three domains, N-terminal DNA binding domain, hinge region and ligand-binding domain. Among these three domains, ligand-binding domain is the most complex one. The ligand-binding domain binds to the calcitriol and causes dimerization of VDR or binding of VDR to retinoid receptor. This causes activation of the vitamin D response element in the DNA (Figure 4). Coregulatory complexes, chromatin remodeling complexes, histone acetylation complexes, and mediator complexes, are activated that lead to differential gene expression. This has the effect of increasing the level of serum calcium by acting on intestinal and kidney epithelial cells and bone cells (Figure 4) [24].

## 6. Bone Diseases in the Elderly

During normal human development, the skeletal system grows in three distinct stages. These three stages are as follows:

  Attainment of maximum bone mass (during childhood and early adulthood).
  Maintenance of bone mass through adulthood.
  Diminishing of bone mass with age.

The maximum bone mass and bone loss are determined by endogenous as well as exogenous factors such as a combination of genetic, endocrine, mechanical, and nutritional factors. Defects in these factors cause bone diseases in the elderly.

Two of the main diseases affecting the bone of adults are osteomalacia and osteoporosis. Gustav Pommer, a German pathologist, first described the differences between osteomalacia and osteoporosis using histologic differences in the late nineteenth century. Osteomalacia is the rickets equivalent in adults that results from a deficiency in vitamin D, calcium, or phosphorous. It is characterized by softening of bones. It is not a very common disease and occurs in adults that do not get sufficient exposure to sunlight and adults with malabsorption of calcium and other minerals. In this paper, we will not be extensively discussing this disease. Osteoporosis, on the other hand, is a very common disease especially in the elderly. In fact, it is more common than heart attack, stroke, and breast cancer combined. More than 200 million people worldwide suffer from this disease. It is more common in women (1 out of 3 occurrence) than in men (1 out of 5 occurrence). More Whites and Asians suffer from this disease than Africans. Osteoporosis literally means porous bones and results from a higher bone loss that bones gain during remodeling. It often fails to show any symptoms until resulting in a fracture. Risk factors of osteoporosis are deficiency of sex steroid, calcium, and vitamin D, genetics, lack of exercise, high dose of corticosteroids, smoking, alcohol, low body mass, and rheumatoid arthritis [25]. In this paper, we will further consider the mechanism of osteoporosis due to hormone and calcium metabolisms.

## 7. Effect of Aging on Vitamin D and Calcium Metabolism

**Figure 5:**
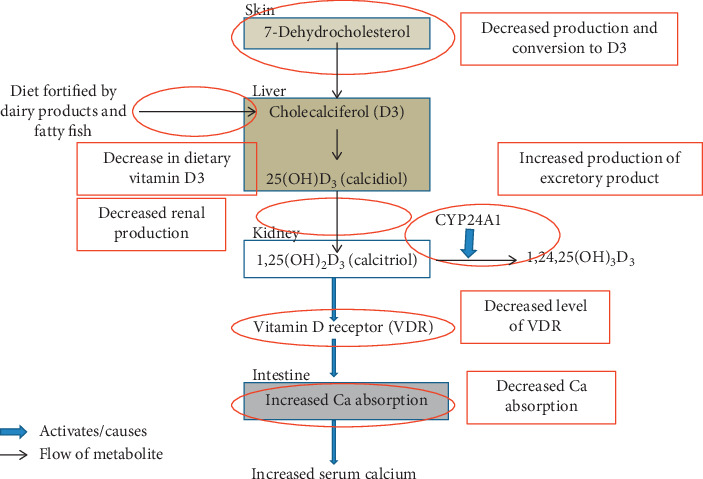
Points along the vitamin D and calcium metabolism pathway affected by age.

Earlier, the metabolism of vitamin D and calcium was described as pathways. There are certain points along the pathway that can be affected by age. We have described six such locations in the pathway below (Figure 5). In the text below, we discuss experiments that try to implicate calcium and vitamin D metabolism to age-related bone loss.

Contrary to the often cited linkage between a decrease in vitamin D with aging and age-related osteoporosis, Tsai et al. have shown that there was no association between serum levels of 25(OH)D_2_, 25(OH)D_3_, or total 25(OH)D_3_ and BMD (bone mineral density) for the three skeletal sites (mid-radius, distal radius, and lumbar spine) [26]. This study was conducted in 122 women aged 33 to 94 years selected randomly in Rochester, MN. Thus, the authors have concluded that in northern American population, reduced bioavailability of vitamin D does not play a major role in age-related osteoporosis [26].

### 7.1. Decreased Production and Conversion to D3

MacLaughlin and Holick conducted a study where they evaluated surgically obtained skin from people aged 8 to 92 years and showed that the skin produced from older individuals had lower concentration of provitamin D3 (7-dehydrocholesterol). The skin of older individuals and younger individuals was subjected to UV light, and the concentration of 7-dehydrocholesterol was measured. It was found that younger individuals had 2-fold more provitamin D than older individuals. This shows that the skin's capacity to produce vitamin D decreases with age [27].

### 7.2. Decrease in Dietary Vitamin D3

A deficiency in vitamin D intake from diet magnifies deficiency in age-related vitamin metabolism as the substrate supply for 25(OH)D_3_ and ultimately 1,25 (OH)₂D_3_ diminishes. Although normally the elderly take vitamin D supplements and do not demonstrate vitamin D deficiency in the blood, Bouillon and Lissens's study in Belgium showed a deficiency of vitamin D substrate and hormone in elderly population. If such deficiency exists in the current world, it is imperative to recognize since it is easily preventable and treatable. The deficiency may be either from diet or from lack of sunlight, and the decrease in serum 25(OH)D_3_ further limits 1,25 (OH)₂D_3_ production, especially during renal dysfunction [28].

### 7.3. Decreased Renal Function

There is a decrease in the activity of renal enzyme 1 *α*-hydroxylase (that converts 25(OH)D_3_ into 1,25 (OH)₂D_3_) as the age increases. Declining renal function was observed when serum 1, 25 (OH)₂D_3_ and serum 25(OH)D_3_ were measured in elderly people. A study conducted in women aged 80–95 who resided in nursing homes demonstrated that they had normal 25(OH)D_3_ levels, but the level of serum 1,25 (OH)₂D_3_ was much lower than in women aged between 65–75 years [29]. Since the level of the precursor is fine but the level of calcitriol is low, it can be surmised that there is an age-related decline in renal function for the conversion of the precursor to calcitriol. This might further result in decrease in calcium absorption due to a decline in calcitriol levels.

Reduction in calcium metabolism can cause secondary hyperparathyroidism and increased bone reabsorption. Secondary hyperparathyroidism resulting from reduced calcium level stimulates renal production of 1,25 (OH)₂D_3_, and this could burden the kidney until it is no longer able to function [29].

### 7.4. Decreased Level of VDR

Several experiments have been conducted to view the effect of aging in the concentration of VDR in the intestine and other organs that might cause a change in calcium metabolism in rats [30] as well as in humans. When old and young rat VDRs were assayed in intestines and bones, it was discovered that there was a higher concentration of VDRs in young rats than adult rats. Similarly, receptor upregulation due to 1,25 (OH)₂D_3_ administration was higher in young rats than old rats [30]. In one of the studies, 321 individuals demonstrated a decrease in the intestinal VDR concentration at older age but showed no change in the serum 1,25 (OH)₂D_3_ concentration [31]. When a similar study was conducted to determine the cause of diminished calcium absorption in the elderly, it was found that there was no significant difference in VDR levels in the intestines of the young and the elderly [32]. Analyzing the results of these three studies, it can be concluded that aging may cause a decline in VDR levels, but the results are not consistent across studies.

### 7.5. Calcium Absorption in the Intestine

A study of age-related decrease of calcium reabsorption was made in postmenopausal women, which showed decrease in calcium level after an age of 75 years. Since the level of serum 1,25 (OH)₂D_3_ and PTH remained unchanged, it can be surmised that the decrease was due to a lack of responsiveness of the small intestine to serum calcitriol. The cause of this decline in intestinal response to calcitriol remains unclear. It can be speculated that the abnormalities lie in the active transport part of calcium since calcitriol stimulates active transport of calcium [33]. Some studies support a reduction in intestinal VDR with age in humans, which might contribute to this age-related intestinal resistance to 1,25 (OH)₂D_3_. We have discussed this in the earlier section.

Contrary to the study in humans, an old study performed in rats showed that the decreased calcium transport with the elderly rats is due to low levels of 1,25 (OH)₂D_3_ in the blood and the intestinal mucosa of rats [34]. This decrease in calcium transport was ameliorated by administration of 1,25 (OH)₂D_3_.

### 7.6. Increased Production of Excretory Product

It has been shown that with increasing age, there is an increase in the activity of CYP24A1, which causes a decrease in active calcitriol levels [35, 36].

## 8. Effect of Aging on PTH Function

It is known that parathyroid hormone levels are often elevated during aging. Hyperparathyroidism can generally be classified into two types: primary and secondary. Primary hyperparathyroidism results from defects in the parathyroid gland. Secondary hyperparathyroidism results from elevated levels of the hormones due to secondary reasons not associated with the gland. Persistently low levels of calcitriol and calcium are one of the causes of secondary hyperthyroidism, when low levels of calcium in the blood trigger high level of parathyroid. This often results from problem with the kidneys, when they fail to convert vitamin D3 to calcitriol.

In a study, the level of serum parathyroid hormone and serum vitamin D3 was measured from individuals ranging from the age of 20 to 90 years. Serum calcium levels progressively declined with age and parathyroid hormone increased along with this decline. This increase in parathyroid in the elderly can be called secondary hyperparathyroidism. There was no difference in the levels of these hormones between men and women [37].

## 9. Effect of Menopause on Bone Loss

Effect of aging on bone loss, both trabecular and cortical, has been widely studied. Menopause in women, which results in a loss of estrogen production, is characterized by loss of cortical bone and further acceleration of the loss of trabecular bone. In men, cortical bone loss is accelerated only after the age of 70 years, about two decades later than women [38]. Often bone formation and bone absorption take place at the discrete foci in the bone called basic multicellular units (BMUs). Estrogen inactivates new BMUs from forming and balances formation and resorption of BMUs. When estrogen levels are low, the number of BMUs and resorption particularly increases. This is a result of prolongation of the lifespan of osteoclast from a lack of apoptosis [39]. Additionally, estrogen is thought to increase osteoblast formation, proliferation, function, and differentiation. Bone formation can thus be inhibited by decreased level of estrogen at old age [40].

Right after menopause, there is an accelerated bone loss. This phase most likely results from a loss in ovarian function and can often be prevented by estrogen replacement [41]. Both E1 and E2 levels fall off in the serum after menopause. During the 2- to 4-year transition after menopause, serum level of E2 falls to 10 to 15% of premenopausal levels. This fall is 25 to 35 percent for serum E1 levels [42]. A modest decrease in serum testosterone is also observed after a loss of ovarian function [43]. Accompanying bone resorption increases by 90% after menopause, and bone formation increases only by 45% [44]. The loss of bone results in loss of calcium from bones. However, hypercalcemia in the serum is prevented when urinary calcium excretion is increased and intestinal calcium absorption is decreased.

To study this bone loss due to menopause in a simpler mouse model, ovariectomized (OVX) mice were used. OVX mice display cortical and trabecular bone loss similar to menopausal women [45]. The role of T cells in bone loss in OVX mice has been elucidated by the finding that T cell deficient nude mice do not display bone loss due to the process of removing ovary. Similar effect is observed when mice are depleted of T cell by injecting anti-T cell antibody, by injecting abatacept that blocks T cell costimulation [46], and by using mice that lack CD40 ligand [47]. It is thought that both CD4+ and CD8+ cells are responsible in bone loss in the OVX mice. The role of Tregs in bone loss in OVX mice has also been elucidated clearly. Tregs inhibit the differentiation of monocytes into osteoclasts that are responsible for bone resorption. Thus, Tregs inhibit bone resorption. The signal for inhibition of bone resorption is conveyed by estrogen to the Tregs. Estrogen stimulates the formation of Tregs [48].

Two mechanisms are thought to be responsible for T cell-induced bone loss in OVX mice: production of TNF by T cells and regulatory interaction between T cell and stromal cells, which leads to enhanced production of cytokines responsible of the activation of osteoclastic cells.

## 10. Diagnosis and Treatment of Osteoporosis

Having discussed the mechanisms of age-related effects of calcium, vitamin D, sex steroid, and parathyroid action on the bone, we next discuss the most important diagnosis and treatment options of osteoporosis, the most common age-related bone disease. The diagnosis of osteoporosis is often done after there is an occurrence of fracture. However, since many people are susceptible to the disease, risk assessment is often carried out and medications are prescribed based on risk assessment without clear symptoms.

For risk assessment, bone mineral density (BMD) is often measured. BMD is measured using dual-energy X-ray absorptiometry (DXA). BMD very closely relates to fracture risk and is measured in units called BMD T score. A score below −2.5 is associated with high risk of osteoporosis [49]. BMD score alone is not a satisfactory predictor of osteoporosis. Other clinical factors such as age, sex, and occurrence of previous fracture are considered before a composite index that is used to estimate the risk of osteoporosis is calculated. Fracture Risk Assessment Tool (FRAX) is the most widely used score to predict risk of osteoporosis. FRAX predicts the probability of having a osteoporotic fracture (a composite of hip, spine, forearm, and proximal humerus fracture) in 10 years [50]. Oftentimes before BMD measurements are taken, a FRAX score is calculated. If the FRAX score is below a certain level based on age, lifestyle advice and reassurance is issued. For a higher FRAX score, BMD measurement is carried out before treatment is given. For a still higher FRAX score, immediate treatment is recommended [50].

Two types of drugs, antiresorptive drugs and anabolic drugs, are prescribed to treat osteoporosis [51]. Antiresorptive drugs act on osteoclasts and decrease the number of sites undergoing bone remodeling, thus decreasing bone remodeling and possibly ameliorating the negative imbalance in remodeling. Antiresorptive drugs increase the BMD. Anabolic drugs on the other hand act by affecting bone modeling and remodeling balance. Teriparatide, an anabolic drug, stimulates the bone modeling process, which is prevalent during bone growth into childhood and early adulthood. Similarly, anabolic drugs also increase the number of remodeling sites and cause positive remodeling balance [51].

Pharmacological intervention in osteoporosis causes significant reduction in vertebral, hip, and other fractures. The reduction in fracture risk attained by the medication depends on medication used and the risk factor for fracture such as the FRAX score [51]. Most of the medications used against osteoporosis work on the pathways described earlier in the paper. The most common drugs against osteoporosis are bisphosphonates, which are antiresorptive agents. The risk of fractures is reduced by bisphosphonates like alendronate, risedronate, ibandronate, and zoledronic acid [51]. Bisphosphonates, as the name suggests, contain two phosphate groups and are similar to pyrophosphate. They mimic pyrophosphate and stop the activity of the enzymes that require pyrophosphate. Bisphosphonates coordinate calcium ions and have a tendency to accumulate at the bones. In the bone tissue, they enter osteoclasts and disrupt the resorption process [52].

Other osteoporosis drugs include RANK ligand inhibitor, estrogen and estrogen analogues, estrogen receptor modulator, and parathyroid hormone receptor agonist. Denosumab, a RANK ligand inhibitor and a monoclonal antibody, causes reversible reduction in bone remodeling [53]. It often needs to be taken for a long duration, and upon discontinuation, the symptoms of osteoporosis return. Since postmenopausal women have reduced estrogen levels that can cause osteoporosis, hormonal therapy with estradiol, estropipate, and conjugated estrogen is sometimes used. Estrogen therapy with progestin is documented to cause a decrease in osteoporotic fractures in postmenopausal women [54]. Cardiovascular and other related risks are associated with administering women 10 years into postmenopause with hormone replacement therapy which is often not recommended [55]. Another class of molecules, estrogen receptor modulators, act as both agonists and antagonist to various estrogen receptors in estrogen target tissues [18]. Raloxifene and bazedoxifene are estrogen receptor modulators sometimes used in osteoporosis treatment. These are weak antiresorptive agents, and raloxifene is active against vertebral fractures but not against nonvertebral and hip fractures [56]. Parathyroid hormone receptor agonists are anabolic drugs that are sometimes used to treat osteoporosis. Teriparatide and abaloparatide are examples of drugs of this class. Teriparatide contains active N terminal 34 amino acids of parathyroid hormone that is bioactive. It selectively activates osteoblasts over osteoclasts and stimulates bone growth [57]. Abaloparatide, like teriparatide, is a parathyroid hormone-related protein analogue that has been approved to treat postmenopausal osteoporosis [58].

A pyramid approach for the treatment of bone diseases including osteoporosis has been suggested by the US Surgeon General. At the base of the pyramid lies sufficient intake of calcium and vitamin D and regular exercise. Treating secondary causes of osteoporosis like lack of estradiol lies at the middle of the pyramid. At the top of the pyramid lies the pharmacotherapy we just discussed [59]. Studies that try to link calcium and vitamin D intake and levels to fracture risks have had inconsistent results [60]. It is likely that these nutrients do not have additional benefits over threshold levels. However, individuals with high risk of osteoporosis, especially the elderly, are recommended to take sufficient levels of vitamin D and calcium.
